# Depression and Smoking Cessation: Evidence from a Smoking Cessation Clinic with 1-Year Follow-Up

**DOI:** 10.1007/s12160-016-9869-6

**Published:** 2016-12-29

**Authors:** Lenka Stepankova, Eva Kralikova, Kamila Zvolska, Alexandra Pankova, Petra Ovesna, Milan Blaha, Leonie S Brose

**Affiliations:** 1 Center for Tobacco-Dependent of the 3rd Medical Department—Department of Endocrinology and Metabolism, First Faculty of Medicine, Charles University in Prague and General University Hospital in Prague, Karlovo namesti 32, 128 00 Praha 2, Czech Republic; 2 Institute of Hygiene and Epidemiology, First Faculty of Medicine, Charles University and General University Hospital Prague, Studničkova 7, 128 00 Praha 2, Czech Republic; 3 Institute of Biostatistics and Analyses at the Faculty of Medicine and the Faculty of Science, Masaryk University, Kamenice 126/3, 625 00 Brno, Czech Republic; 4 Psychology and Neuroscience, King’s College London, UK and UK Centre for Tobacco and Alcohol Studies, Institute of Psychiatry, Addictions Sciences Building, 4 Windsor Walk, Denmark Hill, SE5 8BB London, UK

**Keywords:** Smoking cessation, Depression, Cohort study, Preventive health services, Effectiveness, Evidence-based practice

## Abstract

**Background:**

Smoking is more prevalent among people with depression. Depression may make cessation more difficult and cessation may affect depression symptoms.

**Purpose:**

The aims of this study were to assess the associations between (1) baseline depression and 1-year smoking abstinence and (2) abstinence and change in depression.

**Methods:**

Observational study using data collected routinely in a smoking cessation clinic in the Czech Republic from 2008 to 2014. Aim 1: *N* = 3775 patients; 14.3% reported mild and 15.4% moderate/severe baseline depression levels measured using Beck’s Depression Inventory (BDI-II). Logistic regressions assessed if depression level predicted 1-year biochemically verified abstinence while adjusting for patient and treatment characteristics. Aim 2: *N* = 835 patients abstinent at 1 year; change in depression was analysed using Chi-square statistics, *t* test and mixed method analyses of variance.

**Results:**

Rate of abstinence was lower for patients with mild (32.5%, OR = 0.68; 95% CI: 0.54 to 0.87, *p* = 0.002) and moderate/severe depression (25.8%; OR = 0.57, 95% CI: 0.45 to 0.74, *p* < 0.001) compared with patients without depression (40.5%).

Across abstinent patients, the majority with baseline depression reported lower depression levels at follow-up. Overall mean (SD) BDI-II scores improved from 9.2 (8.6) to 5.3 (6.1); t(834) = 14.6, *p* < 0.001. There were significant main effects of time (F(1832) = 880.8, *p* < 0.001, partial η^2^ = 0.51) and baseline depression level (F(2832) = 666.4, *p* < 0.001, partial η^2^ = 0.62) on follow-up depression and a significant depression * time interaction (F(2832) = 296.5, *p* < 0.001, partial η^2^ = 0.42).

**Conclusions:**

In this effective smoking cessation clinic, depression at the start of treatment predicted reduced smoking abstinence 1 year later. Patients abstinent from smoking experienced considerable improvement in depression.

## Introduction

A substantial proportion of all smokers have a history of depression, and among people with depression, smoking prevalence is about twice as high as in the general population [1–3], leading to increased morbidity and premature mortality [4–6].

There are also associations between depression and smoking cessation. Earlier research raised concerns that smoking cessation may lead to an increase in symptoms, recurrence or even emergence of depression (e.g. [7]), which is one reason why clinicians may be reluctant to address smoking in patients with mental health problems [8]. In contrast to these concerns, a recent systematic review and meta-analysis of longitudinal studies found that compared with continuing to smoke, quitting smoking was associated with a significant decrease in depression from baseline to follow-up [9]. This is corroborated by recent findings from smoking cessation practice indicating continuous improvements in depression symptoms over 1 year among those who quit smoking and no change in those who continued to smoke [10]. However, in a longitudinal study in older smokers, quitting smoking had no impact on depression [11]. Others have found changes in depression to vary depending on the stop smoking medication used [12].

Just as cessation may have effects on depression, depression also appears to affect cessation. Past depression has been found to predict reduced success when attempting to quit smoking. A recent review and meta-analysis found that past major depression was associated with a statistically significant, but modest, decrease in both short- and long-term abstinence rates [13]. Having experienced depression and pre-cessation depressed mood have also been found to be associated with increased relapse [14, 15]. In older smokers (not all of whom were attempting to quit), level of depression predicted continued smoking [11], and there is evidence across all ages that the negative effect of depression on cessation is stronger for women [16, 17].

Despite these findings, research on treating tobacco dependence and depression has been described as being in its infancy [18]. One limitation of the extant research on depression as predictor of cessation success is that studies generally looked at smokers with a history of (major) depression. This has led to several calls for more research into the association between current symptoms or current depression, including mild depression [16, 19, 20]. Many studies explicitly exclude smokers using antidepressants; thus, it remains unclear if smokers taking antidepressants respond as well to smoking cessation treatments as other smokers [16].

The aims of the present analysis were to use data from clinical practice to assess (1) the association between baseline level of depression and 1-year smoking abstinence and (2) change in depression from baseline to 1-year follow-up in those who achieved abstinence.

## Methods

### Intervention

The present data were routinely collected as part of smoking cessation treatment at the Center for Tobacco Dependence at the General University Hospital in Prague, Czech Republic. Patients could be referred by their physician or self-refer to the treatment centre. Treatment followed evidence-based guidelines [21, 22] and was tailored to the individual patient’s needs. Treatment consisted of face-to-face counselling and a choice of pharmacotherapy for all patients and was provided by a nurse and a physician; all physicians had completed training courses developed by the Czech Medical chamber or the Tobacco Treatment Specialist Certification Program at the Mayo Clinic, Rochester, Minnesota.

All patients were treated as outpatients, and over the course of a year, each patient visited the clinic several times. The first visit took about 1 h and was used to complete a basic physical examination and to collect data on demographics, smoking characteristics and dependence, quit attempt history and medical history including self-reported mental health problems and completion of the Beck Depression Inventory (BDI-II) [23]. Patients also provided informed consent for their data to be used in research. The second visit took up to 2 h; it included discussion of physical and psychosocial dependence on smoking and nicotine, strategies to reduce exposure to smoking cues and to cope with cravings. Pharmacotherapy options for smoking cessation were discussed and selected cooperatively and a target date to quit smoking was set. Follow-up visits took about 30 min each. The first one was arranged to occur 1 week after the quit date, followed by fortnightly visits during the first 3 months and monthly visits during months 3 to 6. The final follow-up took place 1 year after the actual quit date; the basic physical examination and BDI-II were repeated and total duration of pharmacotherapy for cessation recorded. At all visits, carbon monoxide (CO) was measured, and information on smoking cessation pharmacotherapy and any changes in medical history were recorded.

The treatment did not include any specific treatment for depression or other mental health problems. The counselling/behavioural support was covered by health insurance, but the cost of pharmacotherapy had to be covered by the patient. A full description of the intervention is available at www.slzt.cz/intervention-structure. Previous analysis showed that at 1-year follow-up, 38% of patients were biochemically verified abstinent from smoking [24]. These are very high success rates compared for example with around 8% achieved in UK stop smoking services [25] (which also deliver a combination of behavioural support and pharmacotherapy, albeit usually restricted to a shorter period of time ) and compared with the Mayo clinic which uses a similar treatment model and reported 28% self-reported abstinence at 6 months [26].

### Measures

#### Patient Characteristics

Demographics recorded during the first (pre-treatment) visit include age, gender, education (primary, secondary, higher education) and marital status (married, divorced, widowed, single; collapsed into married versus not married for analyses). Medical history included cigarette dependence measured using the Fagerström Test of Cigarette/Nicotine Dependence (FTCD/FTND) [27, 28]; for analysis, patients were split according to their scores into ‘low’ (0 to 4) and ‘high’ (5, 10, to) dependence. Depression symptoms were assessed using Beck’s Depression Inventory (BDI-II, [23, 29]); for some analyses, patients were categorised into ‘none or minimal’ (scores ≤13), ‘mild’ (14, 19, to) and ‘moderate/severe’ (≥20) depression; moderate and severe were collapsed to avoid small group sizes. Patients also reported if they were currently taking any antidepressant. Other mental health problems (current and history of) were self-reported by the patients and recorded as anxiety, schizophrenia and bipolar disorder. Because frequency of self-reported mental health problems was low, these were combined into a measure of any other mental health problem (yes, no) for analysis.

#### Intervention Characteristics

Intervention characteristics included in the analysis were number of visits during the year and type and length of pharmacotherapy. A range of pharmacotherapy options and combinations are possible; for analysis, these were coded as follows: none, nicotine replacement therapy (NRT) only (mostly a combination of two or more NRT products), varenicline only, bupropion only or bupropion plus NRT, varenicline plus NRT, other combinations and/or use of electronic cigarettes. Use of a non-nicotinised inhaler (‘paipo’) was recorded, but not treated as pharmacotherapy and not taken into account for categorisation. A combination of pharmacotherapy means that a patient has used more than one type during their quit attempt; this was not always concurrently and includes those who used different options sequentially. Length of pharmacotherapy was recorded as number of months for which pharmacotherapy was used.

#### Outcomes

Smoking abstinence at 1-year follow-up was defined as biochemically verified self-reported abstinence. Patients who reported not having smoked more than 5 cigarettes since quit date who recorded concentrations below 10 ppm of carbon monoxide in exhaled air were recorded as abstinent as defined by the Russell Standard [30]. Patients lost to follow-up despite multiple attempts at contact (15%) were recorded as not abstinent [30].

Depression was again assessed at follow-up using the BDI-II. Scores were used as continuous scale or categorised (none/minimal, mild, moderate/severe) as at baseline.

### Sample

Between January 2008 and December 2014, 4415 smokers began treatment at the Centre for Tobacco Dependence. Patients without information on baseline depression were excluded from all analyses (*n* = 998). Patients younger than 16 (*n* = 16) or older than 80 years (*n* = 5) were also excluded.

To address aim 1 (association between baseline level of depression and outcome), patients missing information on age or dependence (*n* = 16) were excluded, leaving *n* = 3380 patients for analyses. An additional analysis included length of pharmacotherapy so that only patients taking any pharmacotherapy could be included (*n* = 2545).

To address aim 2 (change in depression), only patients with information on depression level at baseline and 1-year follow-up were included (*n* = 864). Among patients who were not abstinent at 1 year, depression at follow-up was generally not recorded; so, analysis had to be restricted to abstinent patients (*n* = 835).

### Analysis

Baseline characteristics of the sample, treatment characteristics and 1-year outcomes were described. The association between baseline level of depression and outcome was assessed in logistic regressions of 1-year abstinence onto baseline level of depression (none, mild, moderate/severe); a first bivariate model was followed by the primary model, a multiple logistic regression that included baseline patient characteristics (age, gender, education, marital status, cigarette dependence and other mental health problems) and treatment characteristics (number of visits, type of pharmacotherapy). A further model added an interaction for level of depression and gender. Separate models replaced depression level with antidepressant use at baseline. Finally, a separate analysis added length of pharmacotherapy to the primary model; this was repeated with an interaction term for length of pharmacotherapy and type of pharmacotherapy.

Change in depression in abstinent patients was analysed using both the categorisation into levels and the continuous BDI-II score. The proportions of patients moving from one category to another were described and significance of difference in proportions from baseline to 1-year follow-up tested with Chi-square statistics. Mean BDI-II scores at baseline and follow-up were compared using a paired *t* test. A mixed model ANOVA assessed main effects of depression level and time as well as the interaction of depression level and time on follow-up BDI-II scores. Wilcoxon signed rank tests and sign tests were used to confirm parametric results of differences in BDI-II scores across time for the overall sample and by baseline level of depression. A further mixed model ANOVA assessed main effects of time and pharmacotherapy and the interaction of the two on mean BDI-II scores. All analyses were conducted using SPSS 22.

## Results

### Association Between Baseline Depression and 1-Year Outcome

Baseline characteristics of the sample and treatment characteristics are described in Table 1. Overall, CO-verified 1-year abstinence was 37.1%. Overall, 29.7% of the sample reported at least some level of depression, and 444 patients (13.1%) were taking antidepressants at baseline. Bivariate analysis results indicate that in comparison with patients without depression, patients with mild depression (OR = 0.71; 95% CI: 0.58 to 0.87, *p* = 0.01) were less likely to be abstinent and patients who reported moderate to severe depression were considerably less likely to be abstinent (OR = 0.51; 95% CI: 0.41 to 0.63, *p* < 0.001). These associations remained very similar in adjusted analysis (Table 1). Higher education, being married and lower dependence were also associated with increased abstinence; gender, age and other mental health problems were not significantly associated with abstinence (Table 1). Compared with those not taking any pharmacotherapy, patients taking any type of pharmacotherapy were more likely to be abstinent, with the exception of the small groups of those using a combination of NRT and varenicline or ‘other’ combinations where differences were not significant (Table 1). More visits during the year increased odds of abstinence, particularly having had 5 or more visits was associated with vastly increased odds of abstinence at the 1-year follow-up (Table 1). In the additional model including interaction terms, there was no evidence of an interaction between gender and baseline level of depression (gender * mild depression: OR = 1.23, 95% CI: 0.77 to 1.98, *p* = 0.40; gender * moderate/severe depression: OR = 1.24, 95% CI: 0.76 to 2.04, *p* = 0.39).

**Table 1:** Adjusted associations between patient and treatment characteristics and 1-year smoking abstinence, *N* = 3380, primary model

Baseline patient characteristics and treatment characteristics	*N* (%); M(SD) for age	% abstinent at 1 year	OR (95% CI)	*p*
Level of depression (BDI-II score)				
*None or minimal (≤13)*	2378 (70.4)	40.5	1	ref
*Mild (14, 19, to)*	483 (14.3)	32.5	0.68 (0.54 to 0.87)	0.002
*Moderate/severe (≥20)* ^*1*^	519 (15.4)	25.8	0.57 (0.45 to 0.74)	<0.001
Gender				
*Women*	1671 (49.4)	36.7	1	ref
*Men*	1709 (50.6)	37.5	1.03 (0.87 to 1.22)	0.73
Age (OR per 10-year increase)	42.7 (13.8)	37.1	1.03 (0.97 to 1.10)	0.26
Education				
*Primary or secondary school* ^*2*^	2428 (71.8)	34.4	1	ref
*Higher education*	952 (28.2)	43.9	1.43 (1.20 to 1.71)	<0.001
Marital status				
*single/divorced/widowed*	2134 (63.1)	34.0	1	ref
*married*	1246 (36.9)	42.4	1.22 (1.03 to 1.45)	0.025
Dependence (FTND score)				
*High (5, 10, to)*	2310 (68.3)	36.6	1	ref
*Low (0 to 4)*	1070 (31.7)	38.1	1.22 (1.02 to 1.46)	0.032
Other mental health problem				
*Yes*	256 (7.6)	26.6	1	Ref
*No*	3124 (92.4)	38.0	1.23 (0.88 to 1.72)	0.23
Number of visits				
*Fewer than 3*	1122 (33.2)	13.8	1	ref
*3 to 4*	1029 (30.4)	26.8	1.78 (1.41 to 2.25)	<0.001
*5 or more*	1229 (36.4)	67.0	9.57 (7.59 to 12.08)	<0.001
Type of pharmacotherapy				
*None*	793 (23.5)	13.1	1	ref
*Nicotine replacement therapy (NRT)*	402 (11.9)	35.3	1.93 (1.39 to 2.68)	<0.001
*Varenicline*	1763 (52.2)	46.7	2.39 (1.84 to 3.11)	<0.001
*Bupropion or bupropion + NRT* ^*3*^	126 (3.7)	45.2	2.77 (1.73 to 4.42)	<0.001
*Varenicline + NRT*	165 (4.9)	40.6	1.35 (0.88 to 2.08)	0.167
*Other combinations and/or electronic cigarette* ^*4*^	131 (3.9)	45.8	1.53 (0.97 to 2.41)	0.070

^1^ *n* = 346 with moderate depression (BDI-II score 20–28): 25.7% abstinence; *n* = 173 with severe depression (BDI-II score ≥29): 26.0% abstinence

^2^ *n* = 292 with primary school: 27.7% abstinence: *n* = 2136 with secondary school: 35.3% abstinence

^3^ *n* = 47 with bupropion only: 47% abstinence: *n* = 79 with NRT and bupropion: 44% abstinence

^4^Includes *n* = 80 varenicline + bupropion (no e-cigarette): 45% abstinence; *n* = 21 varenicline + bupropion + NRT (no e-cigarette): 43% abstinence; *n* = 30 e-cigarettes in any combination (*n* = 25) or exclusively (*n* = 5): 50% abstinence

*n* = 72 used ‘paipo’ in any combination (*n* = 60) or exclusively (*n* = 12): 49% abstinence. This was not treated as pharmacotherapy in analysis and not taken into account for categorisation

In the models replacing level of depression with antidepressant use, patients using antidepressants at baseline were marginally less likely to be abstinent at 1 year than those not using antidepressants (32.9% versus 37.7%, OR = 0.81, 95% CI: 0.65 to 0.999, *p* = 0.049). This association was similar in adjusted analysis, albeit with a wider confidence interval crossing 1 (OR = 0.79, 95% CI: 0.62 to 1.01, *p* = 0.061). The associations of other predictors were very similar to those in the primary model.

When adding length of pharmacotherapy to the primary model (Table 2), the results for mild and moderate/severe depression were very similar to each other. Longer use of pharmacotherapy use was associated with increased abstinence. For pharmacotherapy, the reference category in this model was NRT; patients on a combination of NRT and varenicline or using any of the options in the mixed other category were less likely to be abstinent than those using NRT; other differences were not significant. The ORs associated with a higher number of visits were not as large as in the main model, but the number of visits remained a strong predictor of abstinence (Table 2). No significant interaction was found for length of pharmacotherapy * type of pharmacotherapy (all *p* ≥ 0.05).

**Table 2:** Adjusted associations between patient and treatment characteristics, including length of pharmacotherapy treatment and 1-year smoking abstinence, *N* = 2545

Baseline patient characteristics and treatment characteristics	*N* (%); M (SD) for age	% abstinent at 1 year	OR (95% CI)	*p*
Level of depression (BDI-II score)				
*None or minimal (≤13)*	1804 (70.9)	48.2	1	ref
*Mild (14, 19, to)*	360 (14.1)	38.1	0.65 (0.50 to 0.84)	0.001
*Moderate/severe (≥20)*	381 (15.0)	33.9	0.63 (0.48 to 0.83)	0.001
Gender				
*Women*	1260 (49.5)	44.0	1	ref
*Men*	1285 (50.5)	45.2	1.04 (0.86 to 1.25)	0.70
Age (OR per 10-year increase)	42.7 (13.6)	44.6	1.01 (0.94 to 1.08)	0.83
Education				
*Primary or secondary school*	1797 (70.6)	42.0	1	ref
*Higher education*	748 (29.4)	50.9	1.40 (1.15 to 1.71)	0.001
Marital status				
*single/divorced/widowed*	1564 (61.5)	42.0	1	ref
*married*	981 (38.5)	49.2	1.24 (1.03 to 1.51)	0.027
Dependence (FTND score)				
*High/very high (5, 10, to)*	1785 (70.1)	43.8	1	Ref
*Low (0 to 4)*	760 (29.9)	46.6	1.19 (0.97 to 1.46)	0.093
Other mental health problem				
*Yes*	171 (6.7)	33.3	1	ref
*No*	2374 (93.3)	45.4	1.48 (1.01 to 2.16)	0.045
Number of visits				
*Fewer than 3*	509 (20.0)	19.4	1	ref
*3 to 4*	866 (34.0)	28.5	1.55 (1.18 to 2.05)	0.002
*5 or more*	1170 (46.0)	67.5	5.91 (4.47 to 7.83)	<0.001
Type of pharmacotherapy				
*Nicotine replacement therapy (NRT)*	393 (15.4)	34.9	1	ref
*Varenicline*	1733 (68.1)	47.0	1.13 (0.86 to 1.48)	0.38
*Bupropion or bupropion + NRT*	123 (4.8)	46.3	1.29 (0.80 to 2.09)	0.30
*Varenicline + NRT*	165 (6.5)	40.6	0.57 (0.37 to 0.87)	0.009
*Other combinations and/or electronic cigarette*	131 (5.1)	45.8	0.61 (0.39 to 0.97)	0.037
*Length of pharmacotherapy*				
*Under 1 month*	507 (19.9)	23.1	1	ref
*1 to less than 2 months*	610 (24.0)	25.9	1.12 (0.84 to 1.50)	0.44
*2 to less than 3 months*	348 (13.7)	48.3	1.95 (1.41 to 2.69)	<0.001
*3 to less than 6 months*	619 (24.3)	64.6	2.78 (2.06 to 3.76)	<0.001
*6 months and longer*	461 (18.1)	63.6	3.40 (2.48 to 4.65)	<0.001

### Change in Depression

**Fig. 1:**
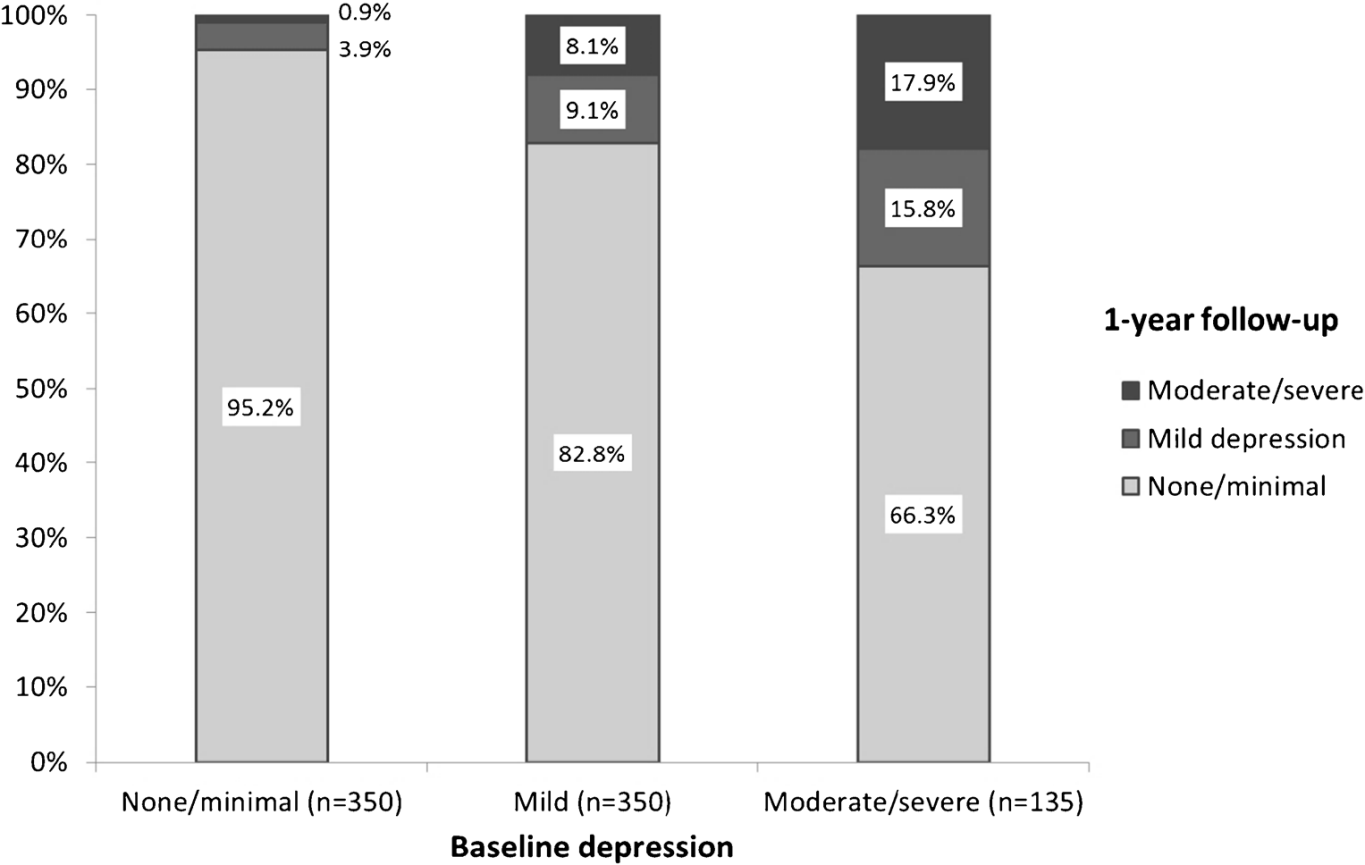
Change in level of depression from baseline to 1-year follow-up among those abstinent at follow-up (*N* = 835)

The majority of patients with depression at baseline who remained abstinent from smoking reported improvements in their level of depression, while only a very small minority reported a higher level of depression at follow-up (Fig. 1, χ^2^ = 100.1, *p* < 0.001). Across all successful patients, mean (SD) BDI-II scores improved significantly from 9.2 (8.6) to 5.3 (6.1); t(834) = 14.6, *p* < 0.001. The median decreased significantly from 7 to 3 (Wilcoxon signed-rank Z = −14.1, *p* < 0.001; sign test Z = −12.9, *p* < 0.001).

**Fig. 2:**
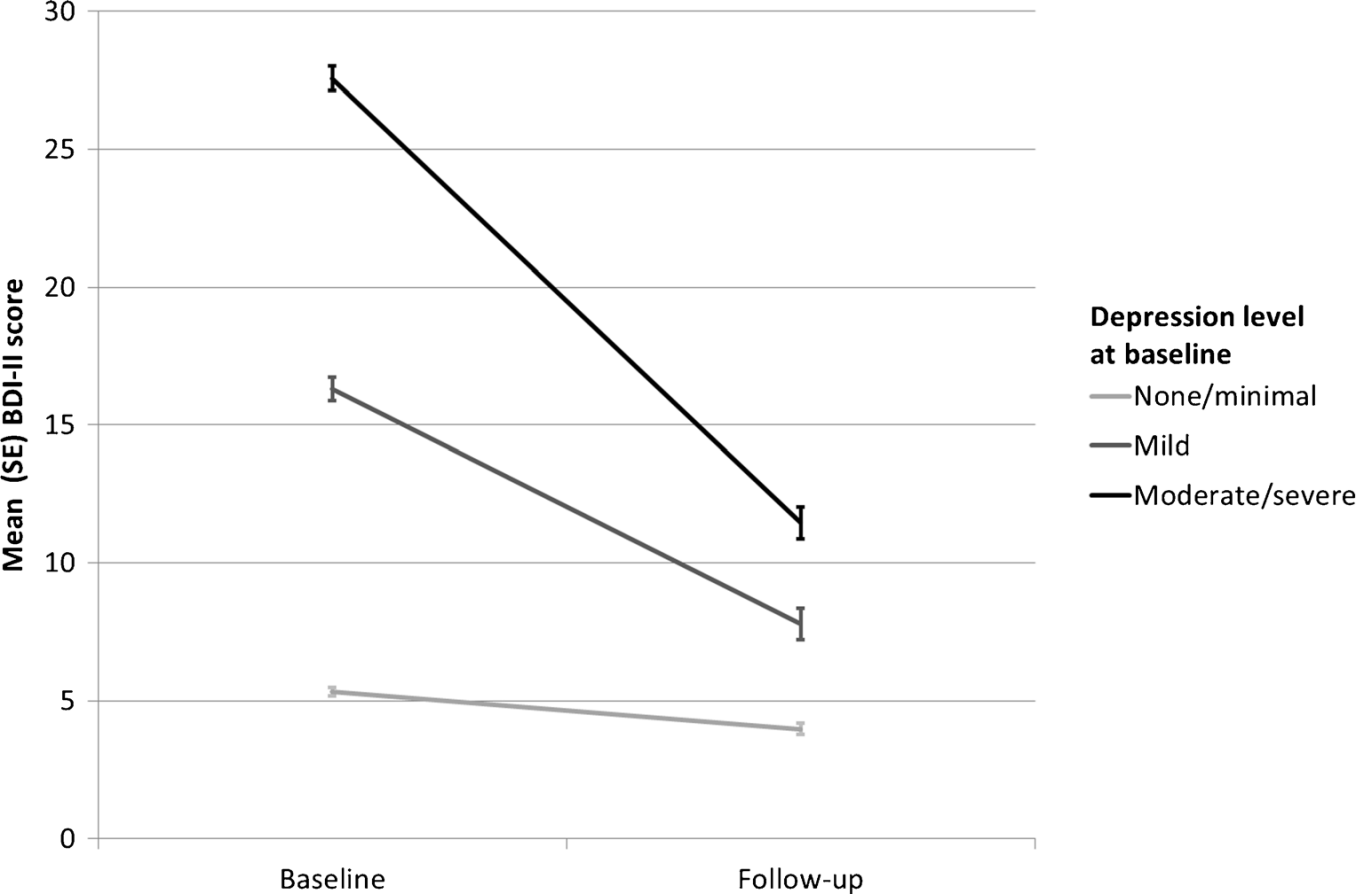
Mean (95%CI) depression (BDI-II) score at baseline and follow-up by baseline level of depression among those abstinent at follow-up (*N* = 835)

The mixed model ANOVA assessing the change in depression by baseline level of depression indicated large significant main effects of time (F(1, 832) = 880.8, *p* < 0.001, partial η^2^ = 0.51) and baseline level of depression (F(2, 832) = 666.4, *p* < 0.001, partial η^2^ = 0.62) and a significant depression * time interaction (F(2832) = 296.5, *p* < 0.001, partial η^2^ = 0.42; Fig. 2). Median scores decreased significantly from baseline to follow-up for those with no/minimal depression (from 5 to 3, Wilcoxon Z = −8.4, sign Z = −8.4, both *p* < 0.001), mild depression (from 16 to 7, Wilcoxon Z = −7.8, sign Z = −7.5, both *p* < 0.001) and moderate/severe depression (from 25 to 10, Wilcoxon Z = −8.1, sign Z = −8.2, both *p* < 0.001).

**Fig. 3:**
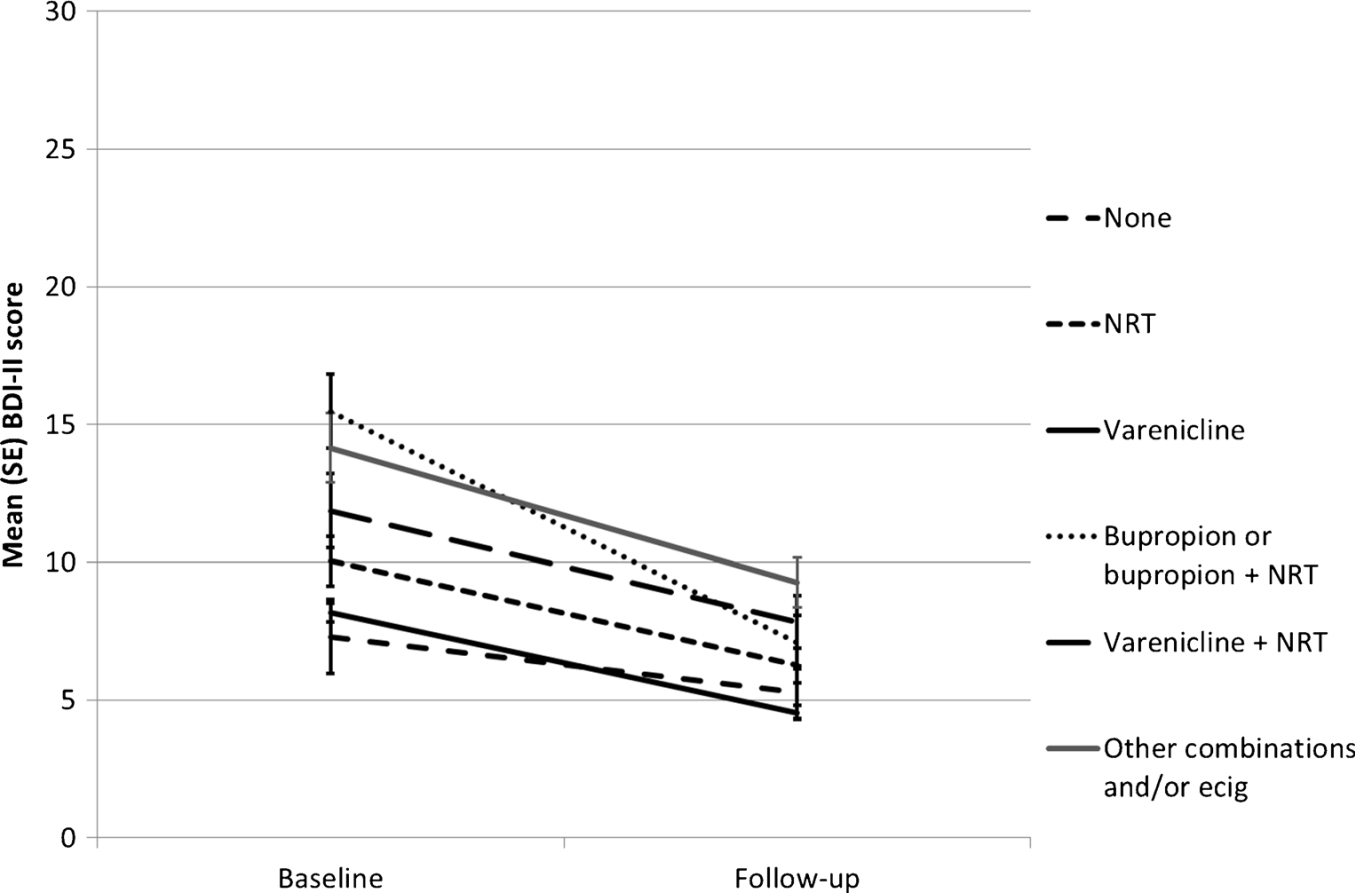
Mean (95%CI) depression (BDI-II) score at baseline and follow-up by pharmacotherapy among those abstinent at follow-up (*N* = 835)

The mixed model ANOVA assessing the change in depression by pharmacotherapy indicated a small significant main effect of pharmacotherapy (F(5, 829) = 12.34, *p* < 0.001, partial η^2^ = 0.07) and a significant interaction of pharmacotherapy with time (F(5, 829) = 3.47, *p* = 0.004, partial η^2^ = 0.02), illustrated by a steep gradient for those on bupropion (Fig. 3).

## Discussion

In a cohort of patients undergoing smoking cessation treatment, patients with depression at the beginning of treatment were less likely to have successfully stopped smoking a year later than patients without depression, and this association remained when adjusting for a number of demographic and treatment characteristics. Those who had stopped successfully reported an improvement in depression; this was true across all levels of depression and those with moderate to severe depression reported the most pronounced improvement.

The reduction in abstinence predicted by depressive symptoms is in line with previous research [12–14, 17]. There was no evidence of differential effects by gender as found in previous studies [16, 17]. As in previous findings on effectiveness [31] and efficacy [32], pharmacotherapy use in this clinic mostly predicted higher abstinence than no medication. This included the antidepressant bupropion for which a recent Cochrane review found only weak evidence of efficacy in smokers with past depression and no clear evidence in smokers with current depression [33]. It is striking that use of pharmacotherapy well beyond the recommended 8 to 12 weeks of use is associated with continuing improvements in abstinence.

The findings on a reduction in depressive symptoms in those who abstain from smoking also support previous findings from a small clinical study [10] and are in line with findings of a meta-analysis [9]. Although reduction differed by medication, we found no evidence of a particularly strong effect of varenicline as previously described [12].

The present findings extend the evidence on the association between depression and subsequent smoking abstinence by focusing on the current level of depression diagnosed using a validated and well-established measure and by including mild depression as well as more severe depression [16]. In an earlier sample from the clinic used in the present study, a history of depression was not associated with abstinence [24], underlining the importance of assessment of depressive symptoms close to the quit attempt. The present study included patients on antidepressants at baseline and found only a very weak association with abstinence, indicating that this is a less sensitive measure than actual depression level. Other strengths include the large sample and that data were routinely collected in clinical practice from a clinic with a high success rate and 1-year follow-up with low attrition rates, thereby extending the evidence base beyond controlled trials and surveys. The present analysis provides further support for the hypothesis that smoking cessation may lead to improvements in depression [9].

Limitations of the present study include that all patients were seen as outpatients, potentially reducing the generalisability to patients so ill that they would be hospitalised. Importantly, the findings on changes in depression have to be considered in the light of the limitation that we were not able to include information on depression at follow-up in those who did not stop smoking and therefore could not compare changes in depression symptoms between those who were and were not abstinent. It is possible that patients who did not stop smoking would have experienced a similar improvement in depression symptoms. Also, depression measures could only be included at two time points, not allowing us to make statements about the direction of the association; it is possible that those who stopped smoking subsequently experienced improvements in depression symptoms or that patients who experienced an improvement in depression were more likely subsequently to stop smoking. However, a recent meta-analysis suggests that the latter explanation is less likely [9].

Future research should assess the change in depression symptoms in smokers who attempt to stop smoking but are not successful. Previous data from small cessation trials [34, 35] and recent data from population surveys suggested that relapse in a quit attempt may predict an increase in depressive symptoms [36].

The steep gradient of success rates linked to the number of visits is notable. Although attenuated by the length of pharmacotherapy use, it remained the strongest predictor of abstinence. In previous analysis of a smaller sample from the clinic, varenicline was associated with higher rates of abstinence even after adjusting for other variables [37]. The difference in odds between these medications in the present adjusted analysis was much smaller, and exploratory post hoc analyses indicated that this was due to the addition of number of visits as predictor. Although the possibility of reverse causality (those being successful more likely to attend subsequent visits) should not be discounted, this suggests that the face-to-face visits contain a high concentration of active ingredients. Future research could aim to code the content of sessions delivered in the clinic using existing taxonomies [38, 39] and assess associations of components with outcomes [40, 41]. This would enable replication of effective components in other health care services and clinics and help improve long-term smoking abstinence in those receiving support.

## Conclusions

In this successful smoking cessation clinic, depression at the start of treatment predicted reduced smoking abstinence at 1 year. The number of treatment sessions attended was a particularly strong predictor of abstinence. Patients who were abstinent from smoking reported considerable improvement in depression and this was true for all levels of depression.
